# The Role of Leaders in Designing Employees’ Work Characteristics: Validation of the Health- and Development-Promoting Leadership Behavior Questionnaire

**DOI:** 10.3389/fpsyg.2019.01049

**Published:** 2019-05-17

**Authors:** Sylvie Vincent-Höper, Maie Stein

**Affiliations:** Department of Work and Organizational Psychology, Universität Hamburg, Hamburg, Germany

**Keywords:** leadership, employee well-being, employee health, health- and development-promoting leadership behavior, measure, validation

## Abstract

In this article, we draw upon the notion that employees’ work characteristics are an important pathway through which leaders influence employee well-being and propose a theoretical framework that integrates perspectives on leadership, occupational stress, and job design. Based on this integrative approach, we developed the health- and development-promoting leadership behavior questionnaire (HDLBQ) for assessing job demands emanating from and job resources provided through the leader. Validation of the measure in German, French, and English using an overall sample of 2,934 employees demonstrated adequate psychometric properties. An examination of the factorial structure revealed three higher-order factors: demanding, development-oriented, and support-oriented leadership. Multigroup confirmatory factor analysis indicated structural equivalence across the three language versions of the HDLBQ. Correlations with employee well-being were moderate, and the HDLBQ explained unique variance in employee well-being beyond that explained by transformational leadership. Suggestions for applications of the HDLBQ and approaches to enhance employee well-being at the workplace are discussed.

## Introduction

Traditionally, effective leadership has been analyzed in terms of employees’ motivation and performance (e.g., Judge and Piccolo, 2004; Judge et al., 2004). However, there is a growing body of research linking leadership with employee well-being (Kuoppala et al., 2008; Skakon et al., 2010). In particular, transformational leadership, i.e., inspirational leadership behaviors that consider employees’ higher-order needs and values (Bass and Riggio, 2006), has been frequently found to contribute to employee well-being (Arnold, 2017).

Yet we know very little about how exactly (transformational) leaders may affect employee well-being (Skakon et al., 2010; Arnold and Connelly, 2013). Recent research indicates that employees’ work characteristics are an important pathway through which leadership is related to employee well-being. Thus, leaders may decrease employees’ demands and enhance their resources, and through this mechanism, they may contribute to employee well-being (Arnold, 2017; Bakker and Demerouti, 2017). Several empirical studies have shown that leaders are able to affect employee well-being by enhancing their work characteristics (e.g., Arnold et al., 2007; Nielsen et al., 2008; Vincent-Höper et al., 2017a). However, the role of leaders as (co-)designers of their employees’ work characteristics has been expanded neither into leadership approaches nor into measures of leadership behavior.

To address this gap, we propose an integrative theoretical framework that combines research on the effects of (a) work characteristics on well-being and (b) leadership on employees’ work characteristics and well-being. Based on this theoretical framework, we developed a measure for assessing demands emanating from and resources provided through the leader. The health- and development-promoting leadership behavior questionnaire (HDLBQ) explicitly recognizes the leader’s ability to influence employees’ levels of demands and resources. The aim of this study is to describe and validate this measure. To establish the validity of the HDLBQ, we seek to evaluate (a) the factorial validity by testing the factorial structure across three language versions (German, French, and English), (b) the construct validity by showing that the HDLBQ is substantially related to employee well-being, (c) the incremental validity by demonstrating that the HDLBQ explains unique variance in employee well-being beyond that explained by transformational leadership.

This article contributes to the existing literature in two important ways. First, we propose a conceptual framework that combines two research areas that have traditionally been investigated separately in occupational health psychology: leadership research and research on work characteristics and well-being (cf. Nielsen et al., 2008). Integrating perspectives on occupational stress, job design, and leadership contributes to the development of a unifying model that enables more rigorous research on the complex interplay among leadership, employees’ work characteristics, and employee well-being. Second, we provide a theory-based and valid tool for assessing leaders’ direct influence on employees’ work characteristics. Investigating leadership behavior that specifically taps employees’ job demands and resources may be a useful approach to obtain an in-depth understanding of the behaviors through which leaders influence employee well-being.

## Theoretical Background

### Defining Employee Well-Being

Well-being is a broad and multidimensional concept (Wright et al., 2017) that not only includes the absence of ill-being (e.g., strain and emotional exhaustion) but also involves positive states (e.g., happiness and work engagement). Though strongly related, positive and negative aspects of well-being are assumed to represent different dimensions instead of two poles on a continuum (Diener, 1984; Rousseau et al., 2008). That is, high levels of positive well-being are not necessarily associated with the absence of symptoms of impaired well-being. Therefore, we adopt a holistic model of employee well-being that takes into account both the negative and the positive components of well-being to acknowledge that leadership behavior might affect the multiple aspects of employee well-being differently (Inceoglu et al., 2018).

### Transformational Leadership and Employee Well-Being

Transformational leadership is the leadership concept that has been most frequently used to examine the relationship between leadership and employee well-being (Vincent-Höper et al., 2017b). Transformational leaders motivate their followers by communicating appealing visions, encourage them to think of different ways of solving problems, recognize their needs, and serve as role models (Bass and Riggio, 2006). Numerous empirical studies have found transformational leadership to be negatively related to employees’ levels of strain (e.g., Gregersen et al., 2014; Holstad et al., 2014), burnout (e.g., Stordeur et al., 2001; Densten, 2005; Kanste et al., 2007), and depressive symptoms (e.g., Munir et al., 2010). Other studies revealed a positive association between transformational leadership with positive well-being (e.g., Arnold et al., 2007; Nielsen et al., 2008).

However, the effect sizes range from low to medium and vary substantially across studies (Nyberg et al., 2005; Montano et al., 2017), suggesting that the relationship might be more complex. Moreover, transformational leadership allows to identify leadership behaviors that specifically promote employee well-being only to a limited extent. That is, the exact contribution of transformational leadership to employee well-being remains unclear. This finding is not surprising because transformational leadership was not developed to predict employee health and well-being. Rather, it was intended to predict employees’ (extra) effort, performance and motivation (Bass, 1985).

### The Impact of Work Characteristics on Employee Well-Being

A large body of occupational stress research provides substantial evidence for the impact of work characteristics on employees’ health and well-being (e.g., Kahn and Byosiere, 1992; Sonnentag and Frese, 2003). According to the job demands-resources (JD-R) model (Bakker and Demerouti, 2007), excessive demands (e.g., work overload and time pressure) and low levels of resources (e.g., job control and social support) are likely to result in health problems. Job demands are defined as the “physical, psychological, social, or organizational aspects of the job that require sustained physical and/or psychological (cognitive and emotional) effort or skills” (Bakker and Demerouti, 2007, p. 312). High levels of job demands are assumed to involve the depletion of employees’ mental and physical capacities, which may result in impaired well-being. Job resources refer to the characteristics of the job that attenuate job demands and the associated negative physical or psychological effects. Moreover, job resources contribute to goal achievement and employees’ personal growth and development (Bakker and Demerouti, 2007).

In several studies, leadership has been investigated as a job resource itself in terms of relationship quality, supervisor support, and supervisory coaching (e.g., Bakker et al., 2005; Xanthopoulou et al., 2007). However, leadership has also recently been discussed as an important determinant of demands and resources in the context of occupational stress models. In their review on the JDR theory, Bakker and Demerouti (2017) suggest that future research should investigate leaders’ impact on employees’ working environment to advance the understanding of processes influencing employee well-being.

### Leadership Behavior and Well-Being: The Mediating Role of Work Characteristics

Several researchers have emphasized the impact of leadership behavior on employees’ perceived levels of demands and resources (e.g., Lobban et al., 1998; Gilbreath and Benson, 2004; Kelloway et al., 2005). Recent research on leadership and employee well-being goes beyond this notion and suggests that employees’ work characteristics are an important pathway through which leaders influence employee well-being (Arnold, 2017; Bakker and Demerouti, 2017).

**FIGURE 1:**
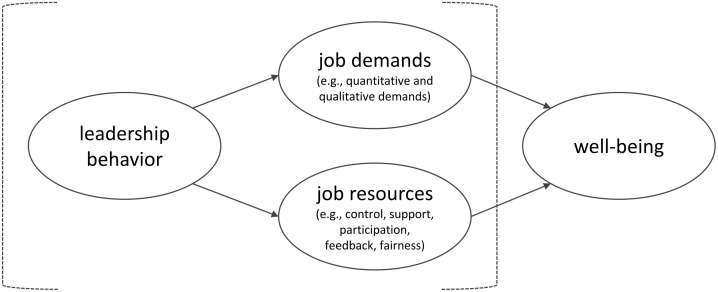
Integrative framework of health- and development-promoting leadership behavior: leaders affect employee well-being through designing employees’ work characteristics.

A considerable body of empirical research has shown that employees’ work characteristics, such as meaningful work (Arnold et al., 2007; Nielsen et al., 2008; Nielsen and Daniels, 2012; Ghadi et al., 2013), perceptions of justice (Walsh et al., 2014), role clarity (Nielsen et al., 2008; Vincent-Höper et al., 2017a), and opportunities for development and growth (Nielsen et al., 2008), mediate the relationship between leadership and employee well-being. In two cross-sectional studies, Fernet et al. (2015) found transformational leadership to be negatively associated with burnout and psychological distress via employees’ perceived levels of cognitive, emotional, and physical work characteristics. In a longitudinal study of 188 elderly care employees, Nielsen et al. (2008) showed that role clarity, meaningful work, and opportunities for development mediated the impact of transformational leadership on positive well-being. While transformational leadership influenced employee well-being via job resources over time, no direct effect of transformational leadership at Time 1 on well-being at Time 2 was found. Thus, transformational leadership may not affect employee well-being “unless it results in changes in perceived work characteristics” (Nielsen et al., 2008, p. 27). We tie in with this notion and incorporate leaders’ ability to shape employees’ work characteristics into an integrative framework that bridges perspectives on occupational stress, job design, and leadership (see Figure 1).

### Measuring Health- and Development-Promoting Leadership Behavior: The Original Version of the HDLBQ

To measure leaders’ impact on employees’ work characteristics, we developed the health- and development-oriented leadership behavior questionnaire (HDLBQ) (Vincent, 2012). The HDLBQ assesses leadership behavior that directly targets employees’ job demands and a variety of job resources. Drawing upon the conservation of resources (COR) theory (Hobfoll, 2001), the HDLBQ focuses on resource-provision through the leader. According to the COR theory, resource loss is the primary process driving psychological strain (Hobfoll, 2001). Moreover, the HDLBQ accounts for the different functions of resources. The JD-R model states that job resources have several different functions: they “(a) are functional in achieving work goals, (b) reduce job demands and the associated physiological and psychological costs, or (c) stimulate personal growth and development” (Bakker et al., 2005, p. 170). Therefore, we included job resources that are expected to play a more supportive role (e.g., instrumental support/information, clarity/transparency) and job resources that may have a stronger effect on employee growth and development (e.g., complexity/variability, control).

#### Item Generation and Item Review

In the first step, the items of the original version of the HDLBQ were generated in German (Vincent, 2012). The selection of job demands and job resources was based on a stepwise approach including both qualitative and quantitative techniques. Based on an extensive review of the existing literature, we identified key work characteristics that have been shown to affect employee well-being (e.g., Kahn and Byosiere, 1992; Sonnentag and Frese, 2003) and that leaders may influence (e.g., Arnold et al., 2007; Nielsen et al., 2008; Thomas and Lankau, 2009). For the generation of items, four groups of 7–12 leaders and employees working in different sectors provided a set of leadership behaviors that contribute to employee well-being and development. Several valid and reliable measures assessing job demands and resources, such as the Job Diagnostics Survey (JDS; Hackman and Oldham, 1975), were used to adapt the wording of the items to fit leadership behaviors. This procedure yielded 23 different categories of health-relevant leadership behaviors, including 146 items in total. To reduce the number of items and examine content validity, 12 researchers in the field of work psychology reviewed the items and rated them by simplicity, clarity, practical relevance, and observability. Moreover, we asked the researchers to classify each item into one of the 23 different categories (Cohen’s kappa coefficient κ = 0.94–0.99). In total, 66 items were selected through this review procedure (Vincent, 2012).

#### Examining the Construct Validity of the Original Version

The factorial structure of the original version of the measure was evaluated using a sample with heterogeneous sociodemographic characteristics that represents a wide range of the target population. Data were collected from 822 employees working in the service sector (14%), health care (14%), manufacturing industry (11%), retail (9%), public services (8%) and other industries in Germany via an online survey. Participants were asked to rate the extent to which their direct supervisor provides job resources and places job demands on them on a five-point response scale ranging from *strongly disagree* to *strongly agree* (Vincent, 2012).

Exploratory factor analysis revealed three higher-order leadership factors: demanding leadership, development-oriented leadership, and support-oriented leadership. Demanding leadership includes both qualitative overload (e.g., delegating overly difficult tasks to employees) and quantitative overload (e.g., delegating too many tasks and putting employees under time pressure). Development-oriented leadership refers to delegating complex tasks that require the use of various skills and showing confidence in employees’ abilities. Moreover, development-oriented leadership is intended to provide control, e.g., when planning and executing tasks, and to encourage participation in decision making. Support-oriented leadership refers to the provision of task-related resources, e.g., clarification of responsibilities, transparency of decisions and goals, adequate information, and instrumental support. Furthermore, support-oriented leaders provide social resources, such as recognition, career support, integrity, fairness, conflict management, and care. The three leadership factors were shown to be substantially related to different indicators of employee well-being, such as emotional exhaustion, psychological strain, psychosomatic complaints, and occupational self-efficacy (Vincent, 2012).

### Development of a More Parsimonious Version of the HDLBQ

When conducting organizational research, survey length is at a premium and using comprehensive, psychometrically sound measures is critical (Stanton et al., 2002). The original version of the HDLBQ included 20 scales with 66 items in total. To develop and evaluate a valid, more parsimonious version of the HDLBQ, we reduced the number of items based on the data used for the validation of the original version (Vincent, 2012).

A common way to select items and reduce scale length is to apply procedures that maximize internal consistency. This approach involves selecting those items with the highest inter-item correlations. However, focusing on maximizing internal consistency may result in reduced construct validity (Clark and Watson, 1995). To avoid restrictions in construct validity, we followed the recommendations of Stanton et al. (2002), who suggest selecting items based on three criteria: judgmental qualities (e.g., subjective judgment of face validity and other non-statistical considerations), internal qualities (e.g., item qualities in reference to the scale), and external qualities (e.g., relations with meaningful external criteria). Therefore, we deleted items that had (a) low loadings on their respective factor, (b) high cross-loadings on other factors, and (c) low correlations with indicators of employee well-being. Moreover, 12 researchers categorized the items into the proposed dimensions of the measure and rated the similarity of the items. Through this procedure, we combined items that were very similar to one another. In addition, we merged strongly correlated scales for which the item classification was ambiguous and that shared the same theoretical background. This approach resulted in a shorter measure of health- and development-promoting leadership behavior, including 14 scales and 35 items in total. Table 1 shows the scales, sample items, and the number of items per scale. We translated the items into French and English and evaluated the accuracy of these versions using the translation-back translation procedure (Brislin, 1970).

**Table 1:** Scales of the HDLBQ with sample items.

Scales	My direct supervisor…	Items
**Demanding leadership**		
Quantitative overload	… often puts me under time pressure.	3
Qualitative overload	… assigns me too much responsibility.	3
**Development-oriented leadership**		
Complexity/variability	… assigns me tasks which require me to use various skills and capabilities.	3
Control	…hands over most of the planning, execution and checking of my work to me.	2
Participation	… takes up my ideas and suggestions.	3
Trust in employees’ abilities	… shows trust in my abilities and actions.	2
**Support-oriented leadership**		
Instrumental support/information	…supports me in the work process when I have difficulties.	3
Clarity/transparency	…clarifies who is responsible for what.	3
Recognition/feedback	… lets me know how well I do my job.	2
Conflict management	… searches for solutions to conflicts with those involved.	2
Cooperation	… encourages the employees to support each other.	2
Career support	… supports the advancement of my career.	2
Integrity/fairness	… is open and honest with me.	3
Care	… asks me about my well-being.	2


### Aims of the Present Study

The objective of the present study is to examine the validity of a more parsimonious version of the HDLBQ across three language versions. To evaluate construct validity, we examine the factorial structure of the measure. We assume that the three higher-order leadership factors (i.e., demanding, development-oriented and support-oriented leadership) found with the original German version of the measure (Vincent, 2012) can be reproduced with the shorter version of the HDLBQ in German, French, and English. Moreover, we assume that the different language versions of the measure do not function differently. Thus, we propose that measurement invariance holds across the three language versions.

To show that the measure is related to meaningful external variables, we examine relationships with multiple indicators of employee well-being. We propose that demanding leadership will be (a) negatively related to positive components of employee well-being and (b) positively related to negative components of employee well-being. Moreover, we hypothesize that support-oriented and development-oriented leadership will be (c) positively related to positive components of employee well-being and (d) negatively related to negative components of employee well-being.

Health- and development-promoting leadership describes specific and observable leadership behaviors. Because the HDLBQ targets work characteristics that are highly relevant to employee well-being, we expect unique contributions from the HDLBQ and propose that health- and development-promoting leadership contribute to employee well-being above and beyond transformational leadership. Therefore, we propose that the measure of health- and development-oriented leadership behavior explains unique variance in all indicators of employee well-being beyond that explained by transformational leadership.

## Materials and Methods

### Participants and Procedure

Participants were recruited from a broad range of industries using an online survey in Germany and France and a paper–pencil survey in the United States. The German sample consisted of 2,242 participants. A total of 70% of the data were collected in seven companies in Germany, 18% in cooperation with a professional survey company and 12% in cooperation with a national association of German engineers. In the seven companies, the response rates ranged from 23 to 80%. Participants worked in the information and technology sector (44%), in the field of engineering, production/fabrication, construction, and research/development (22%), in the retail industry (12%), in public service (5%), in advertisement/media (4%), in transport and logistics (4%), and in the public health sector (3%). The French sample consisted of 386 participants, yielding response rates ranging from 36 to 74%. Participants were recruited in four companies in France and the questionnaire was administered online. Approximately 31% of the participants worked in the telecommunications sector, 26% in the automobile industry, 22% in transport and logistics, and 21% in the pharmaceutical industry. Due to practical requirements (e.g., not all employees had access to a computer at their workplace), we collected the data in the United States sample using a paper–pencil survey. The paper–pencil version of the questionnaire was equivalent to the online version in terms of content, structure, and design. The United States sample consisted of 318 participants working in a transport and logistics company in two branches in California, yielding a response rate of 65%. We excluded 12 participants due to missing data. Thus, the final sample size was *N* = 306. In the German and French samples, there were no missing values because the online survey required the completion of every item. Table 2 shows the characteristics of the three samples. The study was approved by the ethical review committee of the University of Hamburg and informed written consent was obtained from all participants.

**Table 2:** Characteristics of the three samples.

	German version sample	French version sample	English version sample
Sample size (*N*)	2242	386	306
Mean age (*SD*)	39.29 (9.24)	35.83 (8.59)	39.65 (10.20)
Percentage female	52%	44%	41%
Percentage male direct supervisor	77%	73%	77%
Mean duration of employment in years (*SD*)	14.79 (9.59)	13.35 (8.87)	18.87 (11.07)
Mean time with supervisor in years (*SD*)	5.04 (5.28)	4.67 (5.10)	4.48 (4.36)
Mean work hours per week (*SD*)	37.00 (10.63)	38.34 (5.29)	37.53 (8.82)
Percentage managerial position	41%	57%	47%
Percentage college or university degree	60%	70%	80%


*SD, standard deviation.*

### Measures

We measured *health-and development-promoting leadership behavior* using the HDLBQ in its German, French, and English version. Six items of the HDLBQ measure demanding leadership, 19 items support-oriented leadership and 10 items development-oriented leadership. All items were scored on a five-point Likert scale ranging from 1 (*strongly disagree*) to 5 (*strongly agree*). Table 1 displays sample items for each of the subscales. The full version of the HDLBQ in English is available in the Supplementary Material. Table 3 shows Cronbach’s alphas of the scales.

**Table 3:** Means, standard deviations, skewness, kurtosis, Cronbach’s α, and inter-item correlations.

	German version					French version					English version				
			
	*M*	*SD*	Skew	Kurt	α (*r_ii_*)	*M*	*SD*	Skew	Kurt	α (*r_ii_*)	*M*	*SD*	Skew	Kurt	α (*r_ii_*)
QTO	2.26	0.98	0.55 – 0.79	-0.45 – -0.17	0.85	2.60	1.00	0.10 – 0.58	-0.77 – -0.57	0.84	2.64	1.1	-0.06 – 0.53	-1.09 – -0.72	0.85
QLO	1.73	0.75	1.16 – 1.23	1.33 – 1.53	0.84	2.17	0.92	0.46 – 0.92	-0.46 – 0.40	0.85	2.17	1.07	0.66 – 0.86	-0.60 – -0.16	0.90
COV	3.52	0.95	-0.65 – -0.23	-0.71 – -0.09	0.85	3.71	0.90	-0.88 – -0.46	-0.64 – 0.88	0.79	3.78	0.98	-0.98 – -0.67	-0.24 – 0.80	0.85
CON	4.08	0.86	-1.05 – 1.02	0.89 – 0.96	(0.69)	4.08	0.96	-1.21 – -1.18	1.01 – 1.03	(0.71)	3.97	0.97	-1.10 – -0.87	-0.01 – 0.62	(0.56)
PAR	3.54	0.91	-0.52 – -0.41	0.47 – -0.08	0.84	3.65	0.98	-0.86 – -0.54	-0.62 – 0.26	0.82	3.71	1.0	-0.76 – -0.54	-0.71 – -0.02	0.84
TRU	4.13	0.88	-1.17 – -1.03	0.88 – 1.22	(0.78)	4.02	0.93	-1.22 – -1.15	1.20 – 1.51	(0.78)	4.22	0.86	-1.25 – -1.07	0.61 – 1.47	(0.77)
ISI	3.53	0.94	-0.65 – -0.40	-0.39 – -0.27	0.82	3.30	0.98	-0.60 – -0.25	-0.75 – -0.41	0.80	3.58	1.03	-0.73 – 0.05	-0.90 – -0.02	0.77
CTR	3.46	0.98	-0.53 – -0.47	-0.42 – -0.20	0.91	3.46	0.98	-0.68 – -0.39	-0.42 – -0.12	0.86	3.74	1.02	-0.61 – -0.45	-0.59 – -0.27	0.94
REF	3.34	1.08	-0.46 – -0.27	-0.69 – -0.55	(0.76)	3.28	1.06	-0.37 – -0.33	-0.74 – -0.59	(0.73)	3.63	1.06	-0.62 – -0.51	-0.38 – -0.37	(0.79)
CMA	3.50	1.11	-0.55 – -0.49	-0.74 – -0.40	(0.73)	3.10	1.18	-0.32 – -0.16	-0.98 – -0.93	(0.78)	3.44	1.1	-0.46 – -0.31	-0.77 – -0.56	(0.76)
COO	3.60	1.08	-0.61 – -0.57	-0.36 – -0.27	(0.88)	3.30	1.17	-0.42 – -0.38	-0.74 – -0.62	(0.85)	3.72	1.05	-0.65 – -0.59	-0.30 – -0.23	(0.83)
CSU	2.68	1.12	-0.35 – -0.09	-0.85 – -0.76	(0.78)	2.90	1.15	-0.10 – 0.05	-1.01 – -0.99	(0.68)	3.37	1.13	-0.44 – -0.26	-0.82 – -0.62	(0.77)
INF	3.58	0.93	-0.79 – -0.25	-0.50 – 0.08	0.84	3.46	0.96	-0.82 – -0.41	-0.57 – 0.26	0.84	3.66	1.02	-0.73 – -0.08	-0.56 – -0.19	0.82
CAR	3.17	1.12	-0.42 – -0.12	-0.96 – -0.63	(0.74)	3.04	1.12	-0.24 – -0.13	-1.01 – -0.75	(0.67)	3.56	1.1	-0.44 – -0.38	-0.75 – -0.50	(0.82)
															
DL	2.00	0.78	0.75	0.27	(0.64)	2.39	0.91	0.45	-0.28	(0.79)	2.41	1.02	0.41	-0.72	(0.77)
DoL	3.81	0.77	-0.76	0.61	0.87	3.86	0.80	-1.03	1.09	0.87	3.92	0.81	-0.73	0.11	0.86
SoL	3.36	0.86	-0.45	-0.25	0.93	3.23	0.87	-0.53	-0.04	0.92	3.59	0.90	-0.56	-0.09	0.94


*QTO, quantitative overload; QLO, qualitative overload; COV, complexity/variability; CON, control; PAR, participation; TRU, trust in employees’ abilities; ISI, instrumental support/information; CTR, clarity/transparency; REF, recognition/feedback; CMA, conflict management; COO, cooperation; CSU, career support; INF, integrity/fairness; CAR, care; DL, demanding leadership; DoL, development-oriented leadership; SoL, support-oriented leadership. Means (*M*), standard deviations (*SD*), (range of) skewness (skew), and kurtosis (kurt), Cronbach’s α, and inter-item correlations (*r_ii_*). German version sample: *N* = 2,242; French version sample: *N* = 386; English version sample: *N* = 306.*

We assessed *transformational leadership* using the Multifactor Leadership Questionnaire (MLQ-5X; Bass and Avolio, 2000; Felfe, 2006). A sample item is “My supervisor treats me as an individual rather than just a member of a group.” Responses were scored on a five-point Likert scale ranging from 1 (*never*) to 5 (*almost always*). Because the five subscales were highly intercorrelated (up to *r* = 0.85), we computed an overall score of transformational leadership using all 20 items. Cronbach’s alphas were α = 0.96–0.97.

To measure *work engagement*, we used the nine-item Utrecht Work Engagement Scale (UWES; Schaufeli et al., 2006), which is available in German, English, and French. A sample item is, “At work, I feel bursting with energy.” All items were scored on a seven-point rating scale ranging from 1 (*never*) to 7 (*always*). Cronbach’s alphas were α = 0.95 in all samples. We assessed *positive well-being* using three items from the Work Ability Index (WAI) in English (Ilmarinen, 2007) and German (Hasselhorn and Freude, 2005). A sample item is, “Have you recently been active and alert?” Items were scored on a five-point rating scale ranging from 1 (*never*) to 5 (*always*). Cronbach’s alphas were α = 0.78–0.83. We measured *occupational self-efficacy* with six items (Rigotti et al., 2008). A sample item is “Whatever comes my way in my job, I can usually handle it.” Responses were rated on a six-point Likert-scale ranging from 1 (*not at all true*) to 6 (*completely true*). Cronbach’s alphas were α = 0.88–0.92. To assess *perceived strain*, we used eight items measuring irritation, which are available in English and in German (Mohr et al., 2006). A sample item is “I get irritated easily, although I do not want this to happen.” Items were scored on a seven-point scale ranging from 1 (*strongly disagree*) to 7 (*strongly agree*). Cronbach’s alphas were α = 0.91–0.94. We measured *emotional exhaustion* with the nine-item scale from the Maslach Burnout Inventory (MBI) in English (Maslach and Jackson, 1981), French (Dion and Tessier, 1994), and German (Enzmann and Kleiber, 1989). A sample item is “I feel emotionally drained from my work.” The items were scored on a six-point scale ranging from 1 (*several times per year and less*) to 6 (*every day*). Cronbach’s alphas were α = 0.92–0.95. We translated the items measuring transformational leadership, positive wellbeing, occupational self-efficacy, and perceived strain into French using translation-back translation procedure (Brislin, 1970).

### Statistical Analyses

To examine the internal structure of the HDLBQ, we computed confirmatory factor analyses (CFA), which were performed in AMOS Version 18 (Arbuckle, 2014). Three different second-order models were computed using maximum-likelihood estimation. In Model 1, we specified 14 first-order latent factors (leadership behavior scales) that load onto one second-order factor (global leadership factor). Model 2 included 14 first-order factors (leadership behavior scales) that load onto two second-order factors (demanding leadership and resource-oriented leadership). Model 3 specified 14 first-order latent factors and the three second-order factors (demanding leadership, development-oriented leadership, and support-oriented leadership) that were identified in the original version of the instrument (Vincent, 2012). If Model 3 fits the data well and better than Model 1 and Model 2 fit the data, the three second-order factor structure should be favored.

As an indicator of the overall fit of the models, we computed chi-square (χ^2^) statistics. Non-significant chi-square values indicate that the model fits the data well. To compensate for non-normally distributed data and to adjust the chi-square *p*-value, we used the Bollen-Stine bootstrap method (Bollen and Stine, 1992). Because chi-square statistics are sensitive to sample size (Bearden et al., 1982), additional fit indices were considered: the comparative fit index (CFI), squared root mean residual (SRMR), and root mean square error of approximation (RMSEA). General guidelines suggest that values close to 0.95 or higher for CFI, levels of 0.08 or lower for SRMR, and levels of 0.07 or lower for RMSEA indicate adequate fit (Hu and Bentler, 1999; Steiger, 2007).

To evaluate the measurement invariance across the different language versions, we computed multigroup CFA. In Model 1, the factorial structure was constrained to be the same across the groups, but both the first- and the second-order factor loadings were allowed to vary (configural invariance model). In Model 2, factor loadings were constrained to be equal across all groups (metric invariance model). In Model 3, the intercepts of the indicators are constrained to be equal across groups (scalar invariance model). If the change in model fit is negligible, we can accept the model with a higher level of invariance. To compare the fit of the invariance models, we followed recommendations to use the change in CFI (ΔCFI) because the change in the chi-square value performs poorly in evaluating measurement invariance (Cheung and Rensvold, 2002). ΔCFI ≤ 0.01 indicates that two models are equivalent, 0.01 < ΔCFI ≤ 0.02 indicates that equivalence can be assumed, and ΔCFI > 0.02 indicates no equivalence (Vandenberg and Lance, 2000).

To examine if the measure is related to meaningful external variables, correlations with positive and negative indicators of employee well-being were calculated. To evaluate the amount of incremental variance explained beyond that explained by transformational leadership, we conducted hierarchical regression analyses. Correlations and regression analyses were computed using SPSS version 21.

## Results

### Descriptive Statistics

Table 3 displays the descriptive statistics and internal consistencies (Cronbach’s α) of the leadership behavior scales for the three samples. Because Cronbach’s α overestimates the reliability of scales with only two items, inter-item correlations (*r_ii_*) were computed for these scales. All scales show adequate internal consistency levels and inter-item correlations in the three samples. The univariate skewness and kurtosis values of the items were within an acceptable range. Most of the items measuring support-oriented and development-oriented were slightly negatively skewed, while skewness values of the items measuring demanding leadership were positive. Although some items were somewhat skewed (e.g., items measuring qualitative overload and control), the values were not extreme.

### Factorial Validity

**FIGURE 2:**
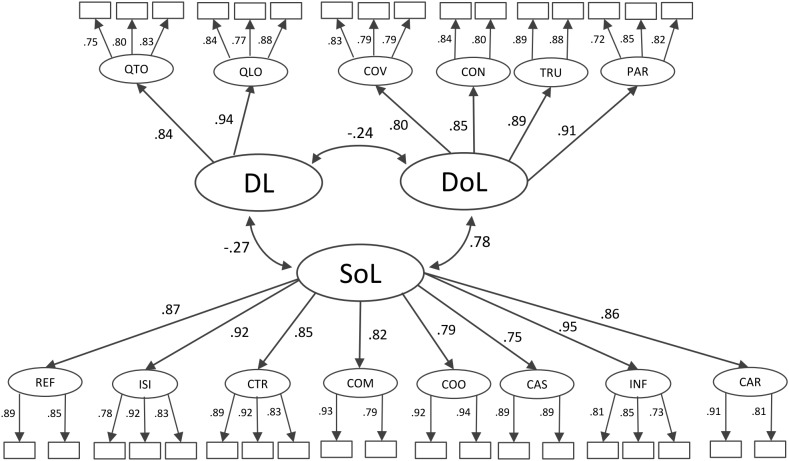
Standardized coefficients of the second-order confirmatory factor structure (Model 3). DL = Demanding Leadership, DoL = Development-oriented Leadership, SoL = Support-oriented Leadership. QTO = Quantitative Overload, QLO = Qualitative Overload, COV = Complexity/Variability, CON = Control, TRU = Trust, PAR = Participation, REF = Recognition/Feedback, ISI = Instrumental Support/Information, CTR = Clarity/Transparency, COM = Conflict Management, COO = Cooperation, CAS = Career Support, INF = Integrity/Fairness, CAR = Care. Items are displayed as rectangles.

Table 4 shows the model fit indices for the different second-order models. The one second-order factor model (Model 1) yielded poor fit with the data in all three samples. The two second-order factor model also showed poor fit with the data in all three samples. For Model 3, fit indices indicate an adequate fit. The chi-square difference tests revealed that the fit of Model 3 was significantly better than that of Model 1 and Model 2. Model 3 comprised 14 first-order latent factors (leadership behavior scales) and three second-order latent leadership factors (demanding leadership, development-oriented leadership, and support-oriented leadership). Figure 2 shows the standardized coefficients of Model 3 for the overall sample. All items loaded substantially onto their respective scales. The correlations between demanding leadership and development- and support-oriented leadership were low, while development-oriented leadership and support-oriented leadership were highly correlated.

**Table 4:** Confirmatory factor analyses.

	Model	χ^2^	df	*p*	SRMR	RMSEA	90% CI RMSEA	CFI	AIC	Δχ^2^
German version	1-factor^a^	6487.695	546	<0.001	0.081	0.070	0.068–0.071	0.899	6655.695	2230.078^∗∗∗^
	2-factor^b^	5590.853	548	<0.001	0.073	0.064	0.063–0.066	0.914	5754.853	1333.236^∗∗∗^
	3-factor^c^	4257.617	546	<0.001	0.063	0.055	0.054–0.057	0.937	4417.228	–
French version	1-factor^a^	2079.645	546	<0.001	0.104	0.085	0.082–0.089	0.841	2247.645	690.536^∗∗∗^
	2-factor^b^	1668.645	548	<0.001	0.081	0.073	0.069–0.077	0.884	1832.645	279.536^∗∗∗^
	3-factor^c^	1389.109	546	<0.001	0.071	0.063	0.059–0.067	0.913	1557.109	–
English version	1-factor^a^	1912.100	546	<0.001	0.105	0.091	0.086–0.095	0.850	2080.100	524.638^∗∗∗^
	2-factor^b^	1619.710	548	<0.001	0.080	0.080	0.076–0.085	0.882	1783.710	232.248^∗∗∗^
	3-factor^c^	1387.462	546	<0.001	0.067	0.071	0.066–0.076	0.908	1555.462	–


*German version sample: *N* = 2,242; French version sample: *N* = 386; English version sample: *N* = 306. SRMR, standardized root mean square residual; RMSEA, root mean square error of approximation; CFI, comparative fit index. ^*a*^ The 14 first-order latent factors (leadership behavior scales) that load onto one second-order factor (global leadership factor). ^*b*^ The 14 first-order factors (leadership behavior scales) that load on two second-order factors (demanding leadership and resource-oriented leadership). ^*c*^ The 14 first-order latent factors and three second-order factors (demanding leadership, development-oriented leadership, and support-oriented leadership). ^∗∗∗^*p* < 0.001.*

### Equivalence of the Three Language Versions

Table 5 shows the fit indices of the configural invariance model and the metric invariance model. The model fit indices indicate acceptable fit for the configural invariance model [χ^2^(1632) = 6970.898, SRMR = 0.061, RMSEA = 0.034, CFI = 0.931] and the metric invariance model [χ^2^(1694) = 7175.965, SRMR = 0.060, RMSEA = 0.033, CFI = 0.929]. ΔCFI was less than 0.01, suggesting metric invariance across the three samples. The model fit for the scalar invariance model was also acceptable [χ^2^(1744) = 8142.429, SRMR = 0.06, RMSEA = 0.035, CFI = 0.918] and ΔCFI was 0.011, indicating that scalar invariance can be assumed across the three samples.

**Table 5:** Model fit indices for the two second-order models to test measurement invariance across the three samples.

Model	χ^2^	df	*p*	SRMR	RMSEA	90% CI RMSEA	CFI	ΔCFI
Model 1: Configural model	6970.898	1632	< 0.001	0.061	0.033	0.033–0.034	0.931	0.002
Model 2: Metric invariance model	7175.965	1694	< 0.001	0.060	0.033	0.032–0.034	0.929	0.011
Model 3: Scalar invariance model	8142.429	1744	< 0.001	0.060	0.035	0.035–0.036	0.918	


*German version sample: *N* = 2,242; French version sample: *N* = 386; English version sample: *N* = 306. Configural model: first- and second-order factor loadings were allowed to vary across groups. Metric invariance model: first- and second-order factor loadings were constrained to be equal across groups. Scalar invariance model: first- and second-order factor loadings and intercepts of the indicators were constrained to be equal across groups.*

### Relationships With Employee Well-Being

As shown in Table 6, the demanding leadership scales (i.e., quantitative and qualitative overload) were negatively related to work engagement, positive well-being, and occupational self-efficacy and were positively related to irritation and emotional exhaustion for all three language versions. The development-oriented and support-oriented leadership scales were positively related to work engagement, positive well-being, and occupational self-efficacy and were negatively related to irritation and emotional exhaustion.

**Table 6:** Correlations between health- and development-promoting leadership behavior and indicators of employee well-being.

	German version sample (*N* = 2,242)					French version sample (*N* = 386)					English version sample (*N* = 306)				
			
	WE^a^	PWB	OSE^a^	IRR	EE	WE	PWB	OSE	IRR	EE	WE	PWB	OSE	IRR	EE
**DL**	-0.14	-0.24	-0.26	0.40	0.46	-0.03	-0.13	-0.02	0.45	0.52	-0.15	-0.14	-0.39	0.45	0.53
Quantitative overload	-0.12	-0.21	-0.17	0.37	0.44	-0.03	-0.15	-0.01	0.46	0.52	-0.11	-0.12	-0.32	0.44	0.52
Qualitative overload	-0.13	-0.21	-0.31	0.36	0.39	-0.03	-0.09	-0.04	0.39	0.47	-0.18	-0.14	-0.42	0.41	0.48
															
**DoL**	0.51	0.42	0.41	-0.16	-0.27	0.51	0.53	0.46	-0.11	-0.31	0.46	0.44	0.41	-0.23	-0.27
Complexity/variability	0.50	0.37	0.35	-0.07	-0.20	0.51	0.50	0.39	-0.06	-0.24	0.46	0.44	0.31	-0.14	-0.25
Control	0.39	0.34	0.37	-0.17	-0.24	0.39	0.39	0.42	-0.09	-0.25	0.29	0.29	0.36	-0.19	-0.20
Participation	0.47	0.37	0.34	-0.13	-0.24	0.43	0.48	0.38	-0.11	-0.28	0.44	0.43	0.32	-0.24	-0.26
Trust in employees’ abilities	0.38	0.35	0.35	-0.20	-0.26	0.42	0.43	0.39	-0.13	-0.29	0.36	0.32	0.42	-0.20	-0.25
															
**SoL**	0.40	0.39	0.26	-0.22	-0.30	0.41	0.48	0.30	-0.18	-0.30	0.52	0.49	0.30	-0.23	-0.35
Instrumental support/information	0.31	0.34	0.24	-0.21	-0.26	0.23	0.33	0.14	-0.21	-0.27	0.43	0.41	0.24	-0.21	-0.32
Clarity/transparency	0.33	0.33	0.23	-0.20	-0.23	0.29	0.34	0.22	-0.20	-0.29	0.43	0.39	0.29	-0.21	-0.30
Recognition/feedback	0.37	0.33	0.24	-0.17	-0.24	0.42	0.46	0.38	-0.09	-0.24	0.46	0.44	0.23	-0.23	-0.32
Conflict management	0.25	0.27	0.19	-0.18	-0.25	0.27	0.34	0.20	-0.12	-0.17	0.37	0.37	0.24	-0.18	-0.27
Cooperation	0.33	0.32	0.21	-0.17	-0.23	0.34	0.39	0.21	-0.18	-0.27	0.44	0.39	0.30	-0.20	-0.28
Career support	0.36	0.30	0.16	-0.09	-0.18	0.34	0.38	0.26	-0.07	-0.18	0.48	0.47	0.24	-0.13	-0.27
Integrity/fairness	0.36	0.37	0.29	-0.25	-0.32	0.33	0.42	0.21	-0.22	-0.31	0.43	0.42	0.23	-0.22	-0.31
Care	0.33	0.31	0.17	-0.19	-0.28	0.40	0.44	0.29	-0.12	-0.23	0.47	0.45	0.25	-0.20	-0.29
Transformational leadership	0.45	0.39	0.27	-0.18	-0.28	0.41	0.46	0.24	-0.12	-0.23	0.45	0.46	0.29	-0.17	-0.26


*^*a*^*N* = 1,320 because these measures were not used in each of the companies in Germany. DL, demanding leadership; DoL, development-oriented leadership; SoL, support-oriented leadership. WE, work engagement; PWB, positive well-being; OSE, occupational self-efficacy; IRR, irritation; EE, emotional exhaustion. Pearson’s *r* ≥ 0.08, *p* < 0.001 (German version sample), *r* ≥ 0.18, *p* < 0.001 (French version sample); *r* ≥ 0.18, *p* < 0.001 (English version sample).*

Table 6 also shows the correlation coefficients of the higher-order leadership factors and well-being. Demanding leadership showed the highest positive correlations with irritation and emotional exhaustion in all three samples (*r* = 0.40 to *r* = 0.53). Development-oriented leadership is strongly and positively related to work engagement, positive well-being, and occupational self-efficacy (*r* = 0.41 to *r* = 0.53). Support-oriented leadership is strongly related to work engagement and positive well-being (*r* = 0.39 to *r* = 0.49) and is substantially negatively correlated with emotional exhaustion (*r* = -0.30 to *r* = -0.35). Transformational leadership was also substantially related to employee well-being. The correlations ranged between *r* = 0.24 and *r* = 0.46 for the positive indicators and between *r* = -0.12 and *r* = -0.28 for the negative indicators of well-being.

### Incremental Validity

We assumed that the HDLBQ explains unique variance in all indicators of employee well-being beyond transformational leadership. To test the incremental validity of the HDLBQ, we computed hierarchical regression analyses using the overall sample. Table 7 shows the results of the hierarchical regression analyses. In the first step, we included several control variables (country, employee sex, supervisor sex, employee age, employee working hours per week, and duration of working together with the current leader). The second step was performed for both transformational leadership (Step 2a) and the three leadership factors from the HDLBQ (Step 2b) to reveal how much variance the different leadership measures explain separately. The analyses indicate that transformational leadership explained an additional 3 to 19% of the variance in the indicators of employee well-being. Demanding leadership, development-oriented leadership, and support-oriented leadership explained an additional 20 to 30% of the variance in employee well-being. Thus, the three higher-order leadership factors explained a higher proportion of variance in all indicators of employee well-being than transformational leadership did. In Step 3a, we added demanding leadership, development-oriented leadership, and support-oriented leadership after accounting for the effects of the sociodemographic variables and transformational leadership (Step 1 and Step 2a) to examine the additional proportion of variance explained by the HDLBQ. Regression analysis shows that the three higher-order leadership factors explained an additional 8 to 23% of the variance in all indicators of well-being beyond transformational leadership.

**Table 7:** Hierarchical regression analyses.

	Work engagement^a^		Positive well-being		Occupational self-efficacy^a^		Irritation		Emotional exhaustion	
					
Variables	β	*ΔR*^2^	β	*ΔR*^2^	β	*ΔR*^2^	β	*ΔR*^2^	β	*ΔR*^2^
Step 1 control variables										
France	0.14^***^		-0.07^*^		-0.01		0.06^*^		0.07^*^	
United States	0.07^*^		-0.09^***^		0.11^***^		0.11^***^		0.14^***^	
Sex (employee)	0.04		0.02		-0.02		0.00		-0.02	
Sex (supervisor)	0.02		0.02		0.01		-0.03		0.03	
Age	0.01		0.00		0.11^***^		-0.13^***^		-0.14^***^	
Working hours	0.14^***^		0.11^***^		0.16^***^		0.10^***^		0.09^***^	
Duration with supervisor	0.02		0.03		-0.01		0.03		0.02	
Adj. *R*^2^	0.03		0.02		0.05		0.03		0.04	

Step 2a		0.17^***^		0.19^***^		0.07^***^		0.03^***^		0.07^***^
TFL	0.42^***^		0.44^***^		0.27^***^		-0.16^***^		-0.27^***^	
Adj. *R*^2^ after controlling for Step 1	0.20		0.21		0.12		0.06		0.11	

Step 2b		0.25^***^		0.26^***^		0.20^***^		0.21^***^		0.30^***^
DL	-0.03		-0.08^***^		-0.18^***^		0.42^***^		0.46^***^	
DoL	0.35^***^		0.28^***^		0.39^***^		0.01		-0.10^***^	
SoL	0.19^***^		0.26^***^		0.00		-0.14^***^		-0.16^***^	
Adj. *R*^2^ after controlling for Step 1	0.28		0.28		0.25		0.24		0.34	

Step 3a		0.08^***^		0.08^***^		0.13^***^		0.18^***^		0.23^***^
DL	-0.03		-0.08^***^		-0.18^***^		0.42^***^		0.46^***^	
DoL	0.34^***^		0.27^***^		0.38^***^		0.00		-0.10^***^	
SoL	0.06		0.14^***^		-0.05		-0.18^***^		-0.16^***^	
Adj. *R*^2^ after controlling for Step 1 and Step 2a	0.29		0.29		0.25		0.24		0.34	

Step 3b		0.01^***^		0.01^***^		0.00		0.00		0.00
TFL	0.18^***^		0.16^***^		0.06		0.06		-0.01	
Adj. *R*^2^ after controlling for Step 1 and Step 2b	0.29		0.29		0.25		0.24		0.34	


*N = 2,934. ^*a*^*N* = 2,012 because these measures were not used in each of the companies in Germany. TFL, transformational leadership; DL, demanding leadership; DoL, development-oriented leadership; SoL, support-oriented leadership. ^∗^*p* < 0.05; ^∗∗∗^*p* ≤ 0.001.*

To test the proportion of unique variance explained by transformational leadership compared to the HDLBQ, we also included transformational leadership into the regression analysis (Step 3b) after accounting for the effects of the sociodemographic variables and the three leadership factors of the HDLBQ. The analysis indicates that transformational leadership explained an additional 1% of the variance in positive well-being and work engagement but no unique variance in the other indicators of employee well-being.

Hierarchical regression analyses were conducted for each of the three language versions separately and yielded similar results. The additional proportion of variance explained in the positive indicators of well-being by the three higher-order leadership factors of the HDLBQ ranged between 6 and 12% for the German version, 8 and 14% for the French version, and 9 and 18% for the English version. In the negative indicators of well-being, the HDLBQ explained an additional 18 to 21% of the variance for the German version, 18 to 27% for the French version, and 14 to 24% for the English version. In contrast, the additional amount of variance explained by transformational leadership ranged between 1 and 2% for work engagement and positive well-being for all three versions. In the other indicators of well-being, transformational leadership did not explain an additional proportion of variance beyond that explained by the HDLBQ.

## Discussion

In this study, we introduced a theoretical framework that combines perspectives on leadership, occupational stress, and job design to obtain an in-depth understanding of the interplay among leadership, employees’ work characteristics, and employee well-being. Based on this integrative framework, we developed and validated a measure that considers leaders to be (co-)designers of employees’ work characteristics and assesses leaders’ influence on employees’ levels of job demands and resources.

The findings show that the HDLBQ is a reliable and valid measure for assessing the impact of leadership behavior on employees’ well-being. CFA provide evidence for the three higher-order factor structure found with the original German version of the HDLBQ (Vincent, 2012), suggesting that the 14 leadership behavior scales constitute three related yet distinct leadership factors: demanding leadership, development-oriented leadership, and support-oriented leadership. Moreover, the factorial structure was equivalent across the German, French, and English versions of the HDLBQ and scalar invariance can be assumed, indicating that the respondents attribute the same meaning to the latent factors and that the levels of the underlying items are equal across the versions.

Substantial relationships with positive and negative indicators of employee well-being provide evidence for the criterion validity of the HDLBQ. Furthermore, the three higher-order leadership factors of the HDLBQ explained incremental variance in employee well-being, after controlling for transformational leadership.

### Theoretical Implications

This article contributes to the literature in two important ways. First, we emphasize that integrating perspectives on leadership, occupational stress, and job design contributes to the development of a unifying model that enables more rigorous research on the link between leadership and employee well-being. A considerable body of research indicates that (transformational) leadership influences employee well-being indirectly through employees’ levels of job demands and job resources (Arnold et al., 2007; Nielsen et al., 2008; Vincent-Höper et al., 2017a). However, this important leverage to enhance employee well-being has not yet been expanded into leadership approaches. We argue that it may not be sufficient to examine employees’ work characteristics as an underlying mechanism explaining why leaders affect employee well-being. Rather, the design of employees’ work characteristics should be considered a key task for leaders to enhance employee well-being. Explicitly recognizing the leader’s role in designing employees’ work characteristics brings leadership and employee well-being closer together and advances the understanding of how exactly leaders may affect employee well-being (cf. Gilbert et al., 2017).

Second, we provide a theory-based and valid tool for assessing leaders’ direct influence on employees’ work characteristics. Investigating leadership behavior that specifically taps employees’ job demands and job resources may be a useful approach to obtain an in-depth understanding of the specific behaviors through which leaders influence employee well-being.

### Practical Implications

It is widely regarded that leadership development may be effective in occupational health intervention (Kelloway and Barling, 2010). The theoretical framework of health- and development-promoting leadership behavior suggests that qualifying leaders to enable them to reduce employees’ demands and enhance their resources may be a promising approach for organizations to achieve and maintain their employees’ well-being. In contrast to transformational leadership, the HDLBQ allows for the definition of specific and observable leadership behaviors that contribute to employee well-being. The focus of leadership interventions aiming at enhancing employee well-being should provide leaders with practical strategies and tools for shaping employees’ work characteristics. The HDLBQ may be used to (a) increase leaders’ awareness for their role as (co-)designers of employees’ work characteristics, (b) reveal work characteristics that leaders may enhance, (c) identify practical approaches to enhancing employees’ work characteristics, and (d) evaluate the effectiveness of interventions that aim to establish health-promoting leadership.

### Limitations and Future Research

Some limitations need to be considered when interpreting the findings. First, the analyses were based on cross-sectional data, which restricts any conclusions about the causality of the effects. Therefore, reverse causal effects of employee well-being on leaders’ perceived impact on employees’ work characteristics are possible (cf. Zapf et al., 1996). For example, employees with low levels of well-being may perceive their leaders as more demanding and less supportive. In addition, employee well-being is also likely to influence leadership behavior because leaders may withdraw from employees experiencing lower levels of well-being to avoid unpleasant interactions with them (van Dierendonck et al., 2004). Thus, leaders’ behavior may not only influence employee well-being, but employee well-being may also influence (employees’ perceptions of) leaders’ impact on employees’ levels of demands and resources (Nielsen et al., 2008). Therefore, we suggest that future studies examine the associations between the HDLBQ and employee well-being using longitudinal designs.

Moreover, analyses were based on single-source and single-method data, as employees assessed both their leader’s behavior and their well-being. Therefore, common method variance may have inflated the relationships (Podsakoff et al., 2003). However, we followed recommendations to mitigate common method bias. In the instructions of the survey, we emphasized that participation was anonymous and explicitly asked the participants to respond accurately. Moreover, we used specific and concise items and physically separated the items of the HDLBQ and the items measuring well-being in the questionnaire. Finally, we used different response scales for the items of the HDLBQ and the items measuring well-being. Nonetheless, future research should take this limitation into account and use multiple sources of information (e.g., leaders and employees), different assessment methods (e.g., surveys, interviews, observations), and multiple indicators of employee well-being (e.g., self-reports, behavioral and physiological measures; Semmer et al., 2003) to examine the impact of health- and development-promoting leadership behavior on employee well-being.

Because the results show that development-oriented leadership and support-oriented leadership are strongly related, one might question whether development-oriented leadership and support-oriented leadership represent two distinct constructs. However, CFA revealed that the three-factor model fit the data better than the two-factor model did. In addition, the correlations between development- and support-oriented leadership and well-being were similar but not identical, suggesting differential relationships between development-oriented leadership and support-oriented leadership and employee well-being.

On a more general level, the factorial structure of the HDLBQ may also indicate that job resources should be distinguished according to their function. According to the JD-R model, job resources “(a) are functional in achieving work goals, (b) reduce job demands and the associated physiological and psychological costs, or (c) stimulate personal growth and development” (Bakker et al., 2005, p. 170). Support-oriented leadership may enhance resources that are particularly instrumental in achieving work goals and reducing job demands and associated negative physical or psychological effects (e.g., instrumental support/information, clarity/transparency, and care). For example, employees may use these resources (directly) to cope efficiently with high levels of job demands. In contrast, development-oriented leadership behavior may target resources that stimulate employees’ growth and development (e.g., complexity/variability, control, trust in employees’ abilities). These resources may challenge employees to extend their skills (Grant et al., 2007), thus contributing to employees’ development and well-being. In line with this notion, we found that development-oriented leadership was more strongly related to occupational self-efficacy as a psychological capacity for development than support-oriented leadership. To shed light on the different roles of support-oriented and development-oriented leadership in predicting employee well-being, future studies should develop a comprehensive theoretical rationale for the differential functions of resources and examine the underlying processes and mechanisms linking resource provision through the leader with employee well-being. On a related note, investigating interactions between support-oriented and development-oriented leadership in relation to employee well-being may contribute to understanding how different constellations of resource provision through the leader influence employee well-being.

Finally, the HDLBQ assesses leaders’ impact on a variety of demands and resources. However, the measure may not comprise all relevant work characteristics. For example, one might argue that several other demands (e.g., emotional demands, role conflict, and physical demands) are also likely to result in impaired well-being (c.f. Crawford et al., 2010). Therefore, we recommend investigating leadership effects on other demands and resources to complement the framework. The critical point for future research is to identify work characteristics (a) that exist in several different jobs, (b) that have an effect on employee well-being, and (c) that leaders are able to influence.

## Conclusion

The present study highlights that integrating perspectives on occupational stress, job design, and leadership into a comprehensive framework is a promising approach to understanding the interplay among leadership, work characteristics, and employee well-being. Drawing upon the notion that leaders are able to shape employees’ work characteristics, we developed a measure of health- and development-promoting leadership behavior that assesses a variety of job demands emanating from and job resources provided through the leader. Based on the findings for content and construct validity, we suggest that the HDLBQ is a useful and valid tool to examine leaders’ impact on employee well-being. The findings not only advance the understanding of how leaders may enhance employee well-being but also emphasize the importance of leaders in determining employees’ work characteristics. Moreover, this integrative perspective offers several approaches to promoting employees’ health and well-being in practice that may be evaluated using the HDLBQ.

## Ethics Statement

This study was carried out in accordance with the recommendations of the German Psychological Society (DGPs) guidelines, Local Ethics Committee of the Faculty of Psychology and Human Movement at the University of Hamburg. The protocol was approved by the Local Ethics Committee of the Faculty of Psychology and Human Movement at the University of Hamburg. All subjects gave written informed consent in accordance with the Declaration of Helsinki.

## Author Contributions

SV-H contributed to the conception and design of the study and wrote the first draft of the manuscript. MS wrote sections of the manuscript. MS and SV-H organized the database, performed the statistical analysis, and contributed to manuscript revision, read and approved the submitted version.

## Conflict of Interest Statement

The authors declare that the research was conducted in the absence of any commercial or financial relationships that could be construed as a potential conflict of interest.
