# Supplementary material for: The Role of Leaders in Designing Employees’ Work Characteristics: Validation of the Health- and Development-Promoting Leadership Behavior Questionnaire
Source: Front Psychol. 2019 May 17;10:1049. doi: 10.3389/fpsyg.2019.01049 (PMC6534042; doi:10.3389/fpsyg.2019.01049)
Supplement: Supplementary file 1 [file Table_1.docx]

Supplementary Material

The Role of Leaders in Designing Employees’ Work Characteristics: Validation of the Health- and Development-Promoting Leadership Behavior Questionnaire

Sylvie Vincent-Höper*, Maie Stein

Department of Work and Organizational Psychology, Universität Hamburg

*** Correspondence:**Sylvie Vincent-Höper, Department of Work and Organizational Psychology, Universität Hamburg Von-Melle-Park 5, 20146 Hamburg, Germany, sylvie.vincent-hoeper@uni-hamburg.de

# Supplementary Tables

*Full version of the HDLBQ in English*

|  | **My direct supervisor ...** | **strongly**  **disagree** | **some- what**  **disagree** | **some-what**  **agree** | **agree** | **strongly**  **agree** |
| --- | --- | --- | --- | --- | --- | --- |
|  |  |  |  |  |  |  |
| QTO1 | … often puts me under time pressure. | ➀ | ➁ | ➂ | ➃ | ➄ |
| QTO2 | … assigns me too many tasks, which overwhelm me because of the time required. | ➀ | ➁ | ➂ | ➃ | ➄ |
| QTO3 | … expects me to work too quickly. | ➀ | ➁ | ➂ | ➃ | ➄ |
| QLO1 | … often gives me tasks which are too difficult and overwhelm me. | ➀ | ➁ | ➂ | ➃ | ➄ |
| QLO2 | … assigns me too much responsibility. | ➀ | ➁ | ➂ | ➃ | ➄ |
| QLO3 | … overwhelms me with tasks which are too complex. | ➀ | ➁ | ➂ | ➃ | ➄ |
| COV1 | ... gives me tasks which allow me to further develop my skills. | ➀ | ➁ | ➂ | ➃ | ➄ |
| COV2 | … gives me the responsibility to make many important decisions. | ➀ | ➁ | ➂ | ➃ | ➄ |
| COV3 | … assigns me tasks which require me to use various skills and capabilities. | ➀ | ➁ | ➂ | ➃ | ➄ |

|  | **My direct supervisor ...** | **strongly**  **disagree** | **some- what**  **disagree** | **some-what**  **agree** | **agree** | **strongly**  **agree** |
| --- | --- | --- | --- | --- | --- | --- |
|  |  |  |  |  |  |  |
| CON1 | ... lets me to decide for myself how I organize my tasks. | ➀ | ➁ | ➂ | ➃ | ➄ |
| CON2 | ... hands over most of the the planning, execution and checking of my work to me. | ➀ | ➁ | ➂ | ➃ | ➄ |
| TRU1 | … shows trust in my abilities and actions. | ➀ | ➁ | ➂ | ➃ | ➄ |
| TRU2 | … trusts me to perform well in an independent and responsible manner. | ➀ | ➁ | ➂ | ➃ | ➄ |
| PAR1 | … makes it possible for me to influence which tasks I handle. | ➀ | ➁ | ➂ | ➃ | ➄ |
| PAR2 | … takes up my ideas and suggestions. | ➀ | ➁ | ➂ | ➃ | ➄ |
| PAR3 | … involves me when planning changes. | ➀ | ➁ | ➂ | ➃ | ➄ |
| REF1 | … gives recognition for personal initiative. | ➀ | ➁ | ➂ | ➃ | ➄ |
| REF2 | … lets me know how well I do my work. | ➀ | ➁ | ➂ | ➃ | ➄ |
| ISI1 | ... is always available to me when I have work-related problems. | ➀ | ➁ | ➂ | ➃ | ➄ |
| ISI2 | ... supports me in the work process when I have difficulties. | ➀ | ➁ | ➂ | ➃ | ➄ |
| ISI3 | ... always provides me with comprehensive information so that I can complete my tasks. | ➀ | ➁ | ➂ | ➃ | ➄ |

|  | **My direct supervisor ...** | **strongly**  **disagree** | **some- what**  **disagree** | **some-what**  **agree** | **agree** | **strongly**  **agree** |
| --- | --- | --- | --- | --- | --- | --- |
|  |  |  |  |  |  |  |
| CTR1 | ... explains the goals to be achieved in an understandable way. | ➀ | ➁ | ➂ | ➃ | ➄ |
| CTR2 | … explains decisions in an understandable way. | ➀ | ➁ | ➂ | ➃ | ➄ |
| CTR3 | ... clarifies who is responsible for what. | ➀ | ➁ | ➂ | ➃ | ➄ |
| CMA1 | … searches for solutions to conflicts with those involved. | ➀ | ➁ | ➂ | ➃ | ➄ |
| CMA2 | ... takes into account the various interests when solving conflicts. | ➀ | ➁ | ➂ | ➃ | ➄ |
| COO1 | … encourages the employees to support each other. | ➀ | ➁ | ➂ | ➃ | ➄ |
| COO2 | … encourages the employees to solve problems together. | ➀ | ➁ | ➂ | ➃ | ➄ |
| CSU1 | … supports the advancement of my career. | ➀ | ➁ | ➂ | ➃ | ➄ |
| CSU2 | … advises me on how I can reach my career goals. | ➀ | ➁ | ➂ | ➃ | ➄ |
| INF1 | ... keeps to arrangements and agreements. | ➀ | ➁ | ➂ | ➃ | ➄ |
| INF2 | … is open and honest with me. | ➀ | ➁ | ➂ | ➃ | ➄ |
| INF3 | … makes sure that the work is fairly distributed among the employees. | ➀ | ➁ | ➂ | ➃ | ➄ |
| CAR1 | … asks me about my well-being. | ➀ | ➁ | ➂ | ➃ | ➄ |
| CAR2 | … encourages me to find a good work-life balance. | ➀ | ➁ | ➂ | ➃ | ➄ |

*Note.* QTO = quantitative overload. QLO= qualitative overload. COV = complexity/variability. CON = control. TRU= trust in employees’ abilities. PAR = participation. REF = recognition/feedback. ISI = instrumental support/ information. CTR = clarity/transparency. CMA = conflict management. COO = cooperation. CSU = career support. INF = integrity/fairness. CAR = care.

The German and the French version of the measure can be obtained from the corresponding author upon request.

*Sample items of the HDLBQ in German and French*

| Scales | Meine direkte Führungskraft / Mon/Ma responsable direct(e)… |
| --- | --- |
| *demanding leadership* |  |
| quantitative overload | … setzt mich häufig unter Zeitdruck. |
|  | … me met souvent sous pression de temps. |
|  |  |
| qualitative overload | … überträgt mir zu viel Verantwortung. |
|  | … me donne trop de responsabilités. |
|  |  |
| *development-oriented leadership* |  |
| complexity/  variability | … überträgt mir Aufgaben, die den Einsatz von vielfältigen Fähigkeiten und Fertigkeiten erfordern. |
|  | … me donne des tâches qui demandent beaucoup de soin et de compétence. |
|  |  |
| control | … überträgt mir weitgehend die Planung, Ausführung und Kontrolle meiner Arbeit. |
|  | … me confie en grande partie la planification, la réalisation et le contrôle de mon travail. |
|  |  |
| participation | … greift meine Ideen und Vorschläge auf. |
|  | … tient compte de mes idées et de mes propositions. |
|  |  |
| trust in employees’ abilities | … zeigt Vertrauen in meine Fähigkeiten und Handlungen. |
|  | … se montre confiant/e dans mes capacités et actions. |
|  |  |
| *support-oriented leadership* |  |
| instrumental support/  information | …unterstützt mich im Arbeitsprozess, wenn ich Schwierigkeiten habe. |
|  | ... me soutient dans les processus de travail, lorsque j’ai des difficultés. |
|  |  |
| clarity/transparency | … sorgt für eindeutige Aufgabenzuständigkeiten und Verantwortlichkeiten. |
|  | … assure la clarté sur les responsabilités relatives à chaque tâche. |
|  |  |
| recognition/feedback | … lässt mich wissen, wie gut ich meine Arbeit mache. |
|  | … me donne des informations sur la qualité de mon travail. |
|  |  |
| conflict management | … sucht bei Konflikten mit den Beteiligten nach Lösungen. |
|  | … cherche une solution aux conflits ensemble avec les personnes concernées. |
|  |  |
| cooperation | … ermutigt die Mitarbeiter, sich gegenseitig zu unterstützen. |
|  | … encourage le personnel, à s’entraider mutuellement. |
|  |  |
| career support | … fördert mein berufliches Vorankommen. |
|  | … appuie ma promotion professionnelle. |
|  |  |
| integrity/  fairness | … geht offen und ehrlich mit mir um. |
|  | … se comporte d’une façons ouverte et honnête envers moi. |
|  |  |
| care | … erkundigt sich nach meinem Wohlergehen. |
|  | … se renseigne sur mon bien-être. |
|  |  |

| *Correlations between the subscales of the HDLBQ in the German sample* | | | | | | | | | | | | | | | | | | | | | | | | | | | |
| --- | --- | --- | --- | --- | --- | --- | --- | --- | --- | --- | --- | --- | --- | --- | --- | --- | --- | --- | --- | --- | --- | --- | --- | --- | --- | --- | --- |
|  |  | 1 |  | 2 |  | 3 |  | 4 |  | 5 |  | 6 |  | 7 |  | 8 |  | 9 |  | 10 |  | 11 |  | 12 |  | 13 |  |
| 1 | quantitative overload |  |  |  |  |  |  |  |  |  |  |  |  |  |  |  |  |  |  |  |  |  |  |  |  |  |  |
| 2 | qualitative overload | .63 | ^***^ |  |  |  |  |  |  |  |  |  |  |  |  |  |  |  |  |  |  |  |  |  |  |  |  |
| 3 | complexity/variability | -.07 | ^**^ | -.01 |  |  |  |  |  |  |  |  |  |  |  |  |  |  |  |  |  |  |  |  |  |  |  |
| 4 | control | -.28 | ^***^ | -.21 | ^***^ | .56 | ^***^ |  |  |  |  |  |  |  |  |  |  |  |  |  |  |  |  |  |  |  |  |
| 5 | participation | -.26 | ^***^ | -.13 | ^***^ | .64 | ^***^ | .65 | ^***^ |  |  |  |  |  |  |  |  |  |  |  |  |  |  |  |  |  |  |
| 6 | trust in employees’ abilities | -.28 | ^***^ | -.24 | ^***^ | .63 | ^***^ | .71 | ^***^ | .63 | ^***^ |  |  |  |  |  |  |  |  |  |  |  |  |  |  |  |  |
| 7 | instrumental support/information | -.35 | ^***^ | -.21 | ^***^ | .47 | ^***^ | .45 | ^***^ | .57 | ^***^ | .57 | ^***^ |  |  |  |  |  |  |  |  |  |  |  |  |  |  |
| 8 | clarity/transparency | -.28 | ^***^ | -.15 | ^***^ | .50 | ^***^ | .46 | ^***^ | .57 | ^***^ | .56 | ^***^ | .76 | ^***^ |  |  |  |  |  |  |  |  |  |  |  |  |
| 9 | recognition/feedback | -.26 | ^***^ | -.12 | ^***^ | .50 | ^***^ | .44 | ^***^ | .57 | ^***^ | .57 | ^***^ | .65 | ^***^ | .63 | ^***^ |  |  |  |  |  |  |  |  |  |  |
| 10 | conflict management | -.31 | ^***^ | -.19 | ^***^ | .38 | ^***^ | .38 | ^***^ | .46 | ^***^ | .48 | ^***^ | .64 | ^***^ | .63 | ^***^ | .59 | ^***^ |  |  |  |  |  |  |  |  |
| 11 | cooperation | -.30 | ^***^ | -.19 | ^***^ | .40 | ^***^ | .42 | ^***^ | .49 | ^***^ | .48 | ^***^ | .62 | ^***^ | .6 | ^***^ | .59 | ^***^ | .66 | ^***^ |  |  |  |  |  |  |
| 12 | career support | -.14 | ^***^ | .03 |  | .53 | ^***^ | .30 | ^***^ | .52 | ^***^ | .43 | ^***^ | .55 | ^***^ | .54 | ^***^ | .66 | ^***^ | .50 | ^***^ | .48 | ^***^ |  |  |  |  |
| 13 | integrity/fairness | -.40 | ^***^ | -.25 | ^***^ | .47 | ^***^ | .49 | ^***^ | .58 | ^***^ | .60 | ^***^ | .76 | ^***^ | .72 | ^***^ | .68 | ^***^ | .71 | ^***^ | .69 | ^***^ | .55 | ^***^ |  |  |
| 14 | care | -.35 | ^***^ | -.14 | ^***^ | .43 | ^***^ | .40 | ^***^ | .53 | ^***^ | .50 | ^***^ | .64 | ^***^ | .60 | ^***^ | .70 | ^***^ | .57 | ^***^ | .59 | ^***^ | .61 | ^***^ | .69 | ^***^ |
|  |  |  |  |  |  |  |  |  |  |  |  |  |  |  |  |  |  |  |  |  |  |  |  |  |  |  |  |
| *Note.* N = 2,242. ** p < .01; *** p < .001 | | | | | | | | | | | | | | | | | | | | | | | | | | | |

| *Correlations between the subscales of the HDLBQ in the French sample* | | | | | | | | | | | | | | | | | | | | | | | | | | | |
| --- | --- | --- | --- | --- | --- | --- | --- | --- | --- | --- | --- | --- | --- | --- | --- | --- | --- | --- | --- | --- | --- | --- | --- | --- | --- | --- | --- |
|  |  | 1 |  | 2 |  | 3 |  | 4 |  | 5 |  | 6 |  | 7 |  | 8 |  | 9 |  | 10 |  | 11 |  | 12 |  | 13 |  |
| 1 | quantitative overload |  |  |  |  |  |  |  |  |  |  |  |  |  |  |  |  |  |  |  |  |  |  |  |  |  |  |
| 2 | qualitative overload | .79 | ^***^ |  |  |  |  |  |  |  |  |  |  |  |  |  |  |  |  |  |  |  |  |  |  |  |  |
| 3 | complexity/variability | -.02 |  | .08 |  |  |  |  |  |  |  |  |  |  |  |  |  |  |  |  |  |  |  |  |  |  |  |
| 4 | control | -.21 | ^***^ | -.14 | ^**^ | .53 | ^***^ |  |  |  |  |  |  |  |  |  |  |  |  |  |  |  |  |  |  |  |  |
| 5 | participation | -.20 | ^***^ | -.07 |  | .59 | ^***^ | .73 | ^***^ |  |  |  |  |  |  |  |  |  |  |  |  |  |  |  |  |  |  |
| 6 | trust in employees’ abilities | -.25 | ^***^ | -.19 | ^***^ | .52 | ^***^ | .65 | ^***^ | .71 | ^***^ |  |  |  |  |  |  |  |  |  |  |  |  |  |  |  |  |
| 7 | instrumental support/information | -.19 | ^***^ | -.60 |  | .40 | ^***^ | .31 | ^***^ | .47 | ^***^ | .46 | ^***^ |  |  |  |  |  |  |  |  |  |  |  |  |  |  |
| 8 | clarity/transparency | -.28 | ^***^ | -.18 | ^***^ | .42 | ^***^ | .41 | ^***^ | .58 | ^***^ | .54 | ^***^ | .67 | ^***^ |  |  |  |  |  |  |  |  |  |  |  |  |
| 9 | recognition/feedback | -.13 | ^**^ | -.05 |  | .49 | ^***^ | .39 | ^***^ | .58 | ^***^ | .55 | ^***^ | .58 | ^***^ | .63 | ^***^ |  |  |  |  |  |  |  |  |  |  |
| 10 | conflict management | -.11 | ^*^ | .01 |  | .32 | ^***^ | .29 | ^***^ | .48 | ^***^ | .41 | ^***^ | .53 | ^***^ | .54 | ^***^ | .53 | ^***^ |  |  |  |  |  |  |  |  |
| 11 | cooperation | -.23 | ^***^ | -.12 | ^*^ | .32 | ^***^ | .29 | ^***^ | .48 | ^***^ | .47 | ^***^ | .59 | ^***^ | .59 | ^***^ | .58 | ^***^ | .61 | ^***^ |  |  |  |  |  |  |
| 12 | career support | -.08 |  | .03 |  | .40 | ^***^ | .33 | ^***^ | .58 | ^***^ | .44 | ^***^ | .53 | ^***^ | .52 | ^***^ | .67 | ^***^ | .59 | ^***^ | .58 | ^***^ |  |  |  |  |
| 13 | integrity/fairness | -.28 | ^***^ | -.14 | ^**^ | .40 | ^***^ | .41 | ^***^ | .57 | ^***^ | .56 | ^***^ | .67 | ^***^ | .69 | ^***^ | .62 | ^***^ | .64 | ^***^ | .72 | ^***^ | .62 | ^***^ |  |  |
| 14 | care | -.18 | ^***^ | -.03 |  | .41 | ^***^ | .37 | ^***^ | .57 | ^***^ | .49 | ^***^ | .57 | ^***^ | .54 | ^***^ | .63 | ^***^ | .59 | ^***^ | .59 | ^***^ | .63 | ^***^ | .70 | ^***^ |
|  |  |  |  |  |  |  |  |  |  |  |  |  |  |  |  |  |  |  |  |  |  |  |  |  |  |  |  |
| *Note*. N = 386. * p < .05; ** p < .01; *** p < .001 | | | | | | | | | | | | | | | | | | | | | | | | | | | |

| *Correlations between the subscales of the HDLBQ in the United States sample* | | | | | | | | | | | | | | | | | | | | | | | | | | | | |
| --- | --- | --- | --- | --- | --- | --- | --- | --- | --- | --- | --- | --- | --- | --- | --- | --- | --- | --- | --- | --- | --- | --- | --- | --- | --- | --- | --- | --- |
|  |  | | 1 |  | 2 |  | 3 |  | 4 |  | 5 |  | 6 |  | 7 |  | 8 |  | 9 |  | 10 |  | 11 |  | 12 |  | 13 |  |
| 1 | quantitative overload | |  |  |  |  |  |  |  |  |  |  |  |  |  |  |  |  |  |  |  |  |  |  |  |  |  |  |
| 2 | qualitative overload | | .77 | ^***^ |  |  |  |  |  |  |  |  |  |  |  |  |  |  |  |  |  |  |  |  |  |  |  |  |
| 3 | complexity/variability | | -.09 |  | -.02 |  |  |  |  |  |  |  |  |  |  |  |  |  |  |  |  |  |  |  |  |  |  |  |
| 4 | control | -.17 | | ^**^ | -.10 |  | .55 | ^***^ |  |  |  |  |  |  |  |  |  |  |  |  |  |  |  |  |  |  |  |  |
| 5 | participation | -.10 | |  | .02 |  | .68 | ^***^ | .68 | ^***^ |  |  |  |  |  |  |  |  |  |  |  |  |  |  |  |  |  |  |
| 6 | trust in employees’ abilities | -.25 | |  | -.22 | ^***^ | .56 | ^***^ | .70 | ^***^ | .65 | ^***^ |  |  |  |  |  |  |  |  |  |  |  |  |  |  |  |  |
| 7 | instrumental support/information | -.19 | | ^***^ | -.06 |  | .53 | ^***^ | .40 | ^***^ | .57 | ^***^ | .53 | ^***^ |  |  |  |  |  |  |  |  |  |  |  |  |  |  |
| 8 | clarity/transparency | -.22 | |  | -.15 | ^**^ | .43 | ^***^ | .37 | ^***^ | .49 | ^***^ | .50 | ^***^ | .68 | ^***^ |  |  |  |  |  |  |  |  |  |  |  |  |
| 9 | recognition/feedback | -.16 | | ^**^ | -.03 |  | .55 | ^***^ | .36 | ^***^ | .61 | ^***^ | .52 | ^***^ | .68 | ^***^ | .67 | ^***^ |  |  |  |  |  |  |  |  |  |  |
| 10 | conflict management | -.18 | | ^**^ | -.08 |  | .40 | ^***^ | .29 | ^***^ | .46 | ^***^ | .41 | ^***^ | .65 | ^***^ | .62 | ^***^ | .60 | ^***^ |  |  |  |  |  |  |  |  |
| 11 | cooperation | -.17 | | ^**^ | -.09 |  | .45 | ^***^ | .30 | ^***^ | .49 | ^***^ | .46 | ^***^ | .66 | ^***^ | .65 | ^***^ | .65 | ^***^ | .70 | ^***^ |  |  |  |  |  |  |
| 12 | career support | -.13 | | ^*^ | .08 |  | .58 | ^***^ | .36 | ^***^ | .61 | ^***^ | .44 | ^***^ | .68 | ^***^ | .57 | ^***^ | .71 | ^***^ | .62 | ^***^ | .61 | ^***^ |  |  |  |  |
| 13 | integrity/fairness | -.24 | | ^***^ | -.08 |  | .48 | ^***^ | .38 | ^***^ | .54 | ^***^ | .52 | ^***^ | .79 | ^***^ | .73 | ^***^ | .71 | ^***^ | .71 | ^***^ | .72 | ^***^ | .70 | ^***^ |  |  |
| 14 | care | -.17 | | ^**^ | -.06 |  | .52 | ^***^ | .38 | ^***^ | .63 | ^***^ | .52 | ^***^ | .72 | ^***^ | .63 | ^***^ | .74 | ^***^ | .6 | ^***^ | .67 | ^***^ | .71 | ^***^ | .76 | ^***^ |
|  |  |  | |  |  |  |  |  |  |  |  |  |  |  |  |  |  |  |  |  |  |  |  |  |  |  |  |  |
| *Note.* N = 306. * p < .05; ** p < .01; *** p < .001 | | | | | | | | | | | | | | | | | | | | | | | | | | | | |
